# Differential analysis of Short chain fatty acids incubation in autistic organoids based on transcriptome sequencing

**DOI:** 10.1371/journal.pone.0351146

**Published:** 2026-06-05

**Authors:** Zaixin Wu, Xinyi Liu, Xiaobo Han, Miao Li

**Affiliations:** School of Mental Health, Jining Medical University, Jining, Shandong, China; Fondazione Don Carlo Gnocchi, ITALY

## Abstract

Autism spectrum disorder (ASD) is characterized by difficulty with social communication and restricted, repetitive patterns of behavior, interest, or activities. We hypothesized that a dysregulation in short-chain fatty acid (SCFA) metabolism induces metabolic dysregulation and proinflammatory responses, which collectively contribute to the social behavioral deficits observed in early childhood. Herein, by high-throughput RNA sequencing (RNA-seq) of the whole transcriptome, including GO and KEGG enrichment analyses, we analyzed global gene expression differences in ASD cerebral organoids exposed to different SCFAs. The ASD cerebral organoids were divided into three groups: the ASD group (control), the acetate-treated group (Z group), and the butyrate-treated group (J group), with three biological replicates per group. Organoids were treated with 100 μM sodium acetate or 100 μM sodium butyrate from day 16 to day 23 of cortical differentiation, for a total duration of 7 days. GO functional annotation revealed that acetate treatment primarily altered gene expression related to differential regulation, whereas butyrate exposure activated immune-related processes. KEGG pathway analysis indicated that butyrate treatment was associated with enrichment of the TGF-β immune-related signaling pathway in ASD organoids, whereas acetate treatment primarily affected molecular functions such as transcriptional regulation, catalytic activity, and membrane permeability.

## Introduction

Autism spectrum disorder (ASD) is a complex neurodevelopmental condition characterized by deficits in social communication and the presence of restricted, repetitive behaviors. Gastrointestinal (GI) disorders are among the most common comorbidities in individuals with ASD, and abnormal short-chain fatty acid (SCFA) profiles have been reported across multiple cohorts. SCFAs, metabolites produced by the gut microbiota, play pivotal roles along the gut–brain axis. In psychiatry, extensive research has explored associations between SCFAs and various mental disorders. In parallel, brain organoids derived from pluripotent stem cells have emerged as powerful models for investigating ASD onset and progression. However, studies specifically addressing SCFA-mediated mechanisms in ASD remain comparatively limited.

ASD typically manifests from the fetal period through early childhood and persists throughout the lifespan once diagnosed, although its definitive etiology remains unclear [1]. Multiple contributing factors have been proposed, including genetic susceptibility, environmental influences, hypoxia, and maternal infection during pregnancy. A large proportion of children with ASD also present GI symptoms, most commonly abdominal distension and constipation. Analyses of the gut microbiota have revealed altered microbial composition and distribution in children with ASD compared with neurotypical controls [2], often characterized by reduced beneficial taxa and increased potentially harmful taxa. Early-life exposures—such as infection, maternal immune activation during pregnancy, and antibiotic use—may disrupt the developing infant gut microbiome and alter its composition. Compared with healthy children, fecal propionate and acetate levels have been reported to be elevated in ASD, whereas butyrate levels are reduced in some cohorts [3]. Other studies likewise report abnormal SCFA profiles in ASD, including lower acetate and butyrate levels [4]. Collectively, these findings suggest that SCFAs may influence host metabolism, intestinal and brain barrier integrity, and immune regulation.

SCFAs are organic acids containing approximately one to six carbon atoms, primarily acetate, propionate, and butyrate, and are generated through microbial fermentation of dietary fiber. They play important roles in energy metabolism, intestinal homeostasis, and immune modulation. Functionally, SCFAs contribute to: (1) **energy supply and metabolic regulation**, with butyrate providing up to 90% of the energy required by colonocytes, while acetate and propionate enter the liver and participate in systemic metabolism [5]; (2) **lipid and glucose regulation**, as propionate inhibits cholesterol synthesis and acetate modulates appetite and lipid storage via gut hormones such as GLP-1 [6]; (3) **maintenance of intestinal barrier integrity**, as butyrate enhances tight-junction protein expression and reduces the risk of increased intestinal permeability [7]; (4) **anti-inflammatory and anti-tumor effects**, including suppression of inflammatory signaling pathways such as NF-κB and induction of apoptosis in aberrant cells; (5) **immune regulation**, through modulation of T-cell differentiation and signaling via G-protein-coupled receptors (e.g., GPR41 and GPR43); and (6) **neuro-metabolic interactions**, as SCFAs can cross the blood–brain barrier and potentially influence brain functions such as cognition and emotion [8].

Traditionally, studies of human brain structure, function, and development have relied on postmortem tissue and animal models. Advances in stem-cell technologies, including embryonic stem cells (ESCs) and induced pluripotent stem cells (iPSCs), have expanded experimental possibilities in neuroscience research [9]. Building on these developments, three-dimensional (3D) organoid systems have attracted growing interest. Lancaster and Knoblich established a 3D culture system from human pluripotent stem cells, termed brain organoids, which recapitulates key cellular compositions and aspects of human brain architecture in vitro [10].

To date, most studies investigating SCFAs in ASD have been clinical in nature, and mechanistic insights remain limited. In the present study, we employ transcriptome sequencing to analyze ASD brain organoids exposed to sodium acetate or sodium butyrate. This approach aims to provide deeper mechanistic insight into gut–brain axis interactions and to characterize SCFA-induced transcriptional changes in an ASD model system.

## Materials and methods

### Sample source and experimental design

The human autism iPSC line GIBHi-001-A was obtained from the Laboratory of the School of Mental Health, Jining Medical University. The establishment and characterization of this autism iPSC line were described previously by Li et al [11]. Organoids were treated from day 16 to day 23 of cortical differentiation, for a total duration of 7 days, with either 100 μM sodium acetate or 100 μM sodium butyrate. The treatment concentrations were selected based on CCK-8 viability assays (**S1 Fig** for acetate; **S2 Fig** for butyrate). For acetate, concentrations ranging from 0 to 1000 μM were tested; 100 μM was selected because it preserved normal cell viability without any toxic drop. For butyrate, 100 μM was chosen as it maintained viability above 90% with no significant cytotoxicity, while avoiding the reduced viability observed at higher concentrations (≥200 μM). Three experimental conditions were established—ASD control, acetate-incubated, and butyrate-incubated—each with three biological replicates, labeled ASD-4, ASD-5, ASD-6; Z-4, Z-5, Z-6; J-4, J-5, J-6, respectively, to ensure reliability and representativeness. This study was approved by the Ethics Committee of Jining Medical University, Jining, 272029.

### RNA-seq library preparation and processing

Total RNA was extracted from the tissue using TRIzol® Reagent according to the manufacturer’s instructions. Then RNA quality was determined by 5300 Bio analyser (Agilent) and quantified using the ND-2000 (NanoDrop Technologies). Only high-quality RNA sample (OD260/280 = 1.8 to 2.2, OD260/230 ≥ 2.0, RQN ≥ 6.5, RIN ≥ 6.3, 28S:18S≥1.0 and RNA quantity >1 μg) was used to construct sequencing library. Libraries were sequenced with the NovaSeq X plus sequencer (2 × 150 bp read length). Adapter-containing reads, reads with N content >10% of the read length, and paired reads in which >50% of bases had Q ≤ 20 were removed to yield high-quality clean reads. All transcriptome analysis for human samples was performed using a reference-based alignment approach. Clean reads were aligned to the human reference genome (GRCh38) using STAR (v2.7.1a), and gene-level expression quantification was performed based on the corresponding GTF annotation file. Differential expression analysis was conducted using DESeq2 [12], with screening thresholds FC ≥ 1 and FDR < 0.05. KEGG enrichment analysis of the filtered DEGs (|log2 fold change| ≥ 1 and FDR < 0.05) was performed, and pathways with adjusted p-values < 0.05 were considered significantly enriched [13,14]. All sequencing data were uploaded to the NCBI SRA database (accession number: SUB15916523) and can be accessed at https://www.ncbi.nlm.nih.gov/sra/.

### Differential expression and enrichment analysis

RNA-seq was performed to profile transcriptomic changes in cortical organoids after short-term exposure to sodium acetate or sodium butyrate. Differentially expressed genes (DEGs) were identified using DESeq2, with thresholds set at |log2 fold change| ≥ 1 and false discovery rate (FDR) < 0.05. GO enrichment analyses were conducted on the identified DEGs, covering Biological Process, Cellular Component, and Molecular Function categories. Pathways with adjusted p-values < 0.05 were considered significantly enriched. The results were interpreted to explore the molecular mechanisms through which SCFA exposure may influence developmental processes in ASD-derived organoids.

## Results

### Selection of SCFA treatment concentrations

To determine appropriate concentrations for acetate and butyrate treatment in ASD organoids, CCK-8 viability assays were performed across a range of concentrations (0, 10, 50, 100, 200, 500, 1000 μM for acetate; 0, 10, 20, 50, 100, 200, 500, 1000 μM for butyrate). As shown in **S1 Fig**, acetate treatment preserved normal cell viability at tested concentrations to 100 μM, with no significant reduction relative to the untreated control. Based on these results, 100 μM acetate was selected for subsequent experiments to ensure sufficient exposure while maintaining optimal cell health. For butyrate (**S2 Fig**), cell viability remained above 90% at concentrations ≤100 μM but declined to approximately 70% at 200 μM and further to 45% at 1000 μM. Therefore, 100 μM butyrate was chosen as the maximum concentration that avoids overt cytotoxicity while allowing potential bioactivity.

### Sequencing quality control and inter-sample correlation

**Fig 1:**
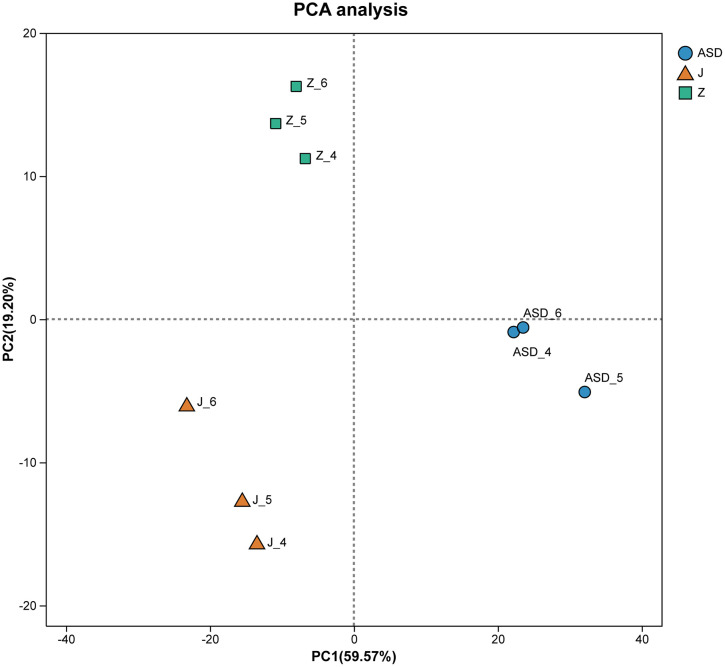
Principal component analysis (PCA) of transcriptomic profiles from ASD-derived cerebral organoids. Each point represents one biological replicate. Blue circles indicate untreated ASD organoids (ASD), green squares indicate ASD organoids treated with sodium acetate (Z), and orange triangles indicate ASD organoids treated with sodium butyrate (J). Organoids were treated with 100 μM sodium acetate or sodium butyrate from day 16 to day 23 of cortical differentiation, for a total duration of 7 days. The first principal component (PC1) explains 59.57% of the total variance, while the second principal component (PC2) explains 19.20% of the variance, demonstrating clear transcriptional separation among the three experimental groups.

Nine transcriptome libraries (three per condition) were generated, yielding a total of 62.49 Gb of clean data. For every sample, clean data exceeded 6.62 Gb, and the proportion of Q30 bases was 95.44%, indicating high read quality. A Spearman correlation matrix was constructed from TPM (transcripts per million) profiles [15]; The distance of each sample point represents the distance between samples. The closer the distance is, the higher the similarity between samples is, and the better the biological reproducibility between samples is. The results showed that ASD group was significantly different from sodium acetate and sodium butyrate treatment groups (**Fig 1**).

### Differential gene expression

**Fig 2:**
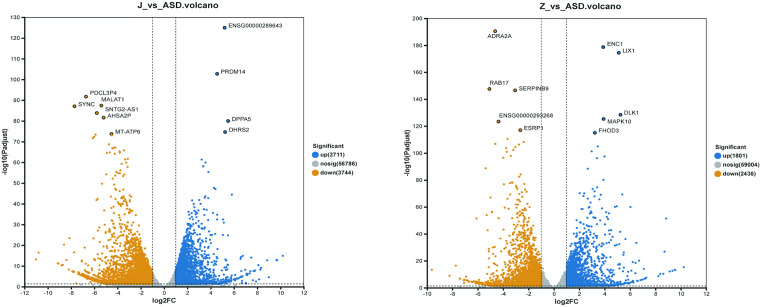
Volcanic map of differentially expressed genes in autism. **(A)** Differentially expressed genes in sodium butyrate–treated ASD cerebral organoids (J) compared with untreated ASD organoids. **(B)** Differentially expressed genes in sodium acetate–treated ASD cerebral organoids (Z) compared with untreated ASD organoids. Each dot represents a gene. Red dots indicate significantly upregulated genes, blue dots indicate significantly downregulated genes, and gray dots represent genes without significant differential expression. Differential expression analysis was performed using DESeq2 with thresholds of |log₂ fold change| ≥ 1 and false discovery rate (FDR) < 0.05. Selected representative genes are labeled.

Based on gene-level quantification, pairwise differential expression analyses were performed with DESeq2, using thresholds |log2FC| ≥ 1 and padjust < 0.05. Relative to the ASD group, the butyrate treated group (J) exhibited 6,455 DEGs, including 2,711 up-regulated and 3,744 down-regulated genes (**Fig 2A**). Compared with ASD, the acetate treated group (Z) showed 4,237 DEGs, comprising 1,801 up-regulated and 2,436 down-regulated genes (**Fig 2B**). These findings indicate that incubation with sodium acetate or sodium butyrate induces extensive transcriptional alterations in ASD organoids.

### GO enrichment analysis of DEGs

**Fig 3:**
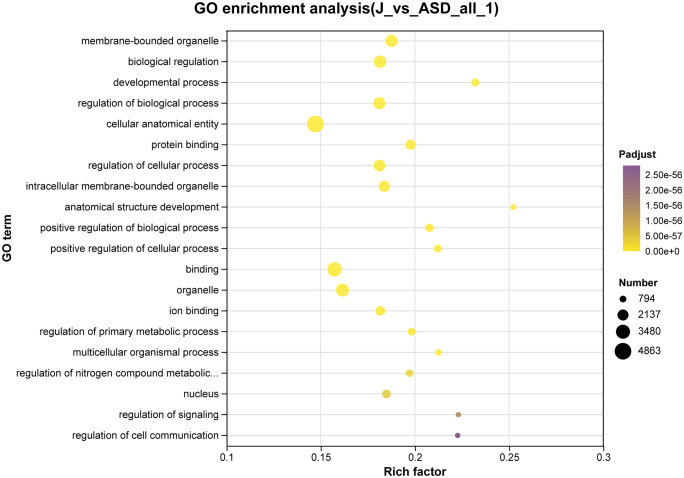
Gene Ontology (GO) enrichment analysis was performed based on differentially expressed genes (DEGs) identified by comparing sodium butyrate–treated ASD cerebral organoids (J) with untreated ASD organoids. DEGs were defined using the criteria |log₂ fold change| ≥ 1 and false discovery rate (FDR) < 0.05. The x-axis represents the rich factor (ratio of DEGs to the total number of genes annotated in each GO term). Dot size indicates the number of genes enriched in each term, and color represents the adjusted p-value (Padjust).

**Fig 4:**
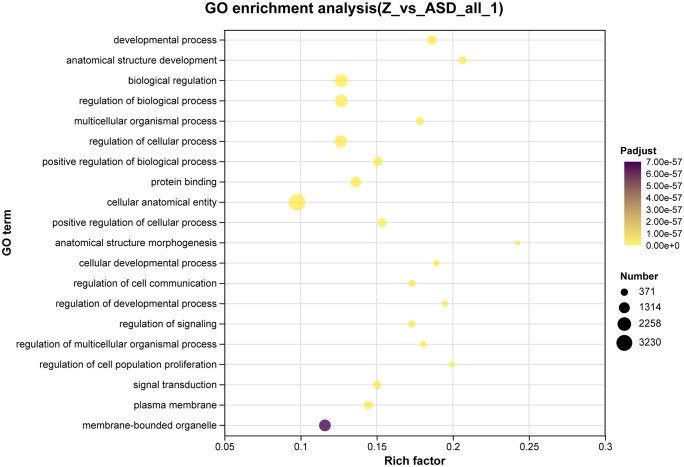
Gene Ontology (GO) enrichment analysis was performed based on differentially expressed genes (DEGs) identified by comparing sodium acetate–treated ASD cerebral organoids (Z) with untreated ASD organoids. DEGs were defined using the criteria |log₂ fold change| ≥ 1 and false discovery rate (FDR) < 0.05. The x-axis indicates the rich factor, dot size reflects the number of enriched genes, and color denotes the adjusted p-value (Padjust).

As shown in **Fig 3**, GO enrichment analysis of DEGs after sodium butyrate incubation revealed, for Biological Process, prominent enrichment in immune-system processes, biological regulation, and metabolic processes; for Cellular Component, enrichments were concentrated in cellular components and organelles; for Molecular Function, DEGs were enriched in catalytic activity, transcription regulator activity, molecular-function regulator activity, and binding. These results suggest a substantial impact of butyrate on immune-related programs in ASD organoids [14]. In contrast, DEGs following sodium acetate incubation (**Fig 4**) were mainly enriched in Molecular Function categories related to regulation of molecular function, transcriptional regulation, catalytic activity, and membrane permeability; in Cellular Component terms associated with membranes and extracellular/membranous components; and in Biological Process terms including multicellular organismal processes, cellular localization, and cellular-component organization.

### KEGG pathway enrichment analysis

**Fig 5:**
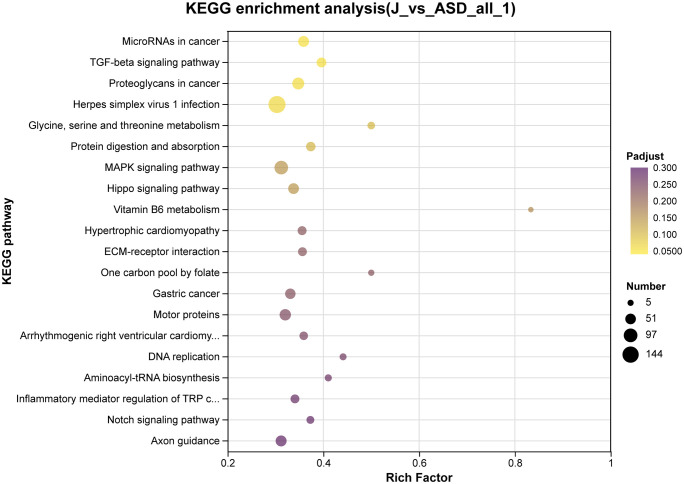
Kyoto Encyclopedia of Genes and Genomes (KEGG) pathway enrichment analysis was performed based on differentially expressed genes (DEGs) identified by comparing sodium butyrate–treated ASD cerebral organoids (J) with untreated ASD organoids. DEGs were defined using the criteria |log₂ fold change| ≥ 1 and false discovery rate (FDR) < 0.05. The x-axis represents the rich factor, dot size corresponds to the number of genes involved in each pathway, and color indicates the adjusted p-value (Padjust).

**Fig 6:**
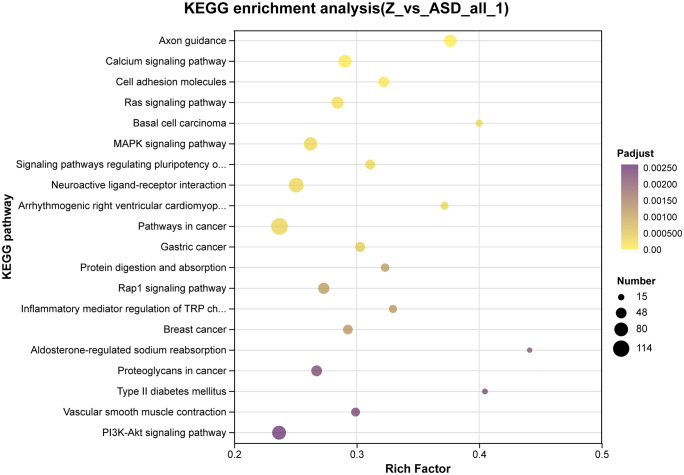
Kyoto Encyclopedia of Genes and Genomes (KEGG) pathway enrichment analysis was performed on differentially expressed genes (DEGs) identified by comparing sodium acetate–treated ASD cerebral organoids (Z) with untreated ASD organoids. DEGs were defined using the criteria |log₂ fold change| ≥ 1 and false discovery rate (FDR) < 0.05. The x-axis indicates the rich factor, dot size represents the number of enriched genes, and color denotes the adjusted p-value (Padjust).

As shown in **Fig 5**, KEGG pathway analysis after sodium butyrate treatment [13] indicated significant enrichment of TGF-β signaling, non-coding RNAs in cancer, glycine/serine/threonine metabolism, protein digestion and absorption, MAPK signaling, and the Hippo signaling pathway. Following sodium acetate incubation, enriched KEGG pathways were primarily axon guidance, calcium signaling, cell adhesion molecules (CAMs), Ras signaling, basal cell carcinoma, MAPK signaling, signaling pathways regulating pluripotency of stem cells, neuroactive ligand–receptor interaction, arrhythmogenic right ventricular cardiomyopathy, pathways in cancer, protein digestion and absorption, and PI3K–Akt signaling (**Fig 6**) [14].

## Discussion

Research on short-chain fatty acids (SCFAs) and autism spectrum disorder (ASD) in China has primarily focused on clinical observations and has generated valuable insights. At the omics level, most studies have examined gut microbiota composition and microbial metabolites. Dysbiosis and SCFA imbalance are frequently reported in individuals with ASD, although findings are not fully consistent across cohorts [3]. Functionally, emerging evidence suggests that supplementation with specific SCFAs may exert beneficial effects in certain contexts. For example, acetate supplementation has been shown to rescue social deficits in Shank3-deficient mice, accompanied by transcriptomic remodeling in the prefrontal cortex, and this effect appears to be independent of microbiota status. In parallel, sodium butyrate has been reported to improve mitochondrial function in patients with ASD [16]. In addition, microbial metabolites have been shown to modulate social behavior by influencing neuronal activity in specific brain regions, such as the bed nucleus of the stria terminalis [17]. Together, these findings provide increasing mechanistic evidence that shifts the role of SCFAs in ASD-related social deficits from a primarily correlative association toward a more causal interpretation.

Despite these advances, the molecular mechanisms through which acetate and butyrate exert their effects in ASD remain incompletely understood. In the present study, we generated brain organoids from ASD-derived induced pluripotent stem cells (iPSCs) and exposed them to sodium acetate or sodium butyrate. Transcriptome sequencing was then applied to identify potential molecular targets of SCFAs, providing a foundation for further mechanistic investigation into how these metabolites may act in ASD.

It is important to acknowledge that, although brain organoids offer unique opportunities to model early human neurodevelopment and cellular diversity [18], their application in psychiatric research remains subject to several limitations. Current organoid systems generally lack functional vasculature and immune components, such as microglia, which constrains their ability to recapitulate neuroinflammatory processes. In addition, the electrophysiological complexity and maturation of organoid neural networks remain limited compared with the adult human brain [19]. Batch-to-batch variability and challenges in standardization further complicate their use in disease modeling [20]. Accordingly, our findings will require validation in more complex experimental systems, including assembloids or in vivo models. Future integration of vascularized and immunocompetent organoids, as well as multi-organ-on-a-chip platforms, may enable a more comprehensive investigation of gut–brain axis mechanisms mediated by microbial metabolites [18,20].

Our transcriptomic analyses revealed significant differences in mRNA expression between untreated ASD organoids and those incubated with sodium butyrate, including genes closely associated with ASD pathophysiology. Previous studies have reported elevated plasma levels of TNF-β1 and TNF-α in children with ASD [21], while reduced TGF-β1 levels have been linked to increased behavioral symptom severity [22]. Consistent with an immunomodulatory role, we observed upregulation of the TGF-β signaling pathway following butyrate treatment in ASD organoids. Given that TGF-β signaling is involved in immune homeostasis, neuronal development, and synaptic plasticity [23], this transcriptional change suggests that butyrate may modulate pathways relevant to ASD pathophysiology. However, whether this effect is specific to ASD organoids or represents a general response to butyrate in neural tissue cannot be determined from the current dataset, as no healthy control organoids were included. Therefore, our findings should be interpreted as hypothesis-generating rather than conclusive about a beneficial effect in ASD.

Following sodium acetate incubation, differentially enriched pathways were predominantly associated with cellular permeability and membrane-related processes. Given that SCFAs are known to regulate epithelial barrier integrity [24], these findings raise the possibility that acetate may influence permeability-related functions in neural cells. However, without comparative data from healthy control organoids, it remains unclear whether this response is characteristic of ASD organoids or reflects a general cellular response to acetate exposure. In addition, microRNAs (miRNAs), which play key roles in post-transcriptional gene regulation, have been implicated in a range of psychiatric disorders, including ASD. However, the mechanistic links between SCFAs, miRNA regulation, and neurodevelopment remain poorly defined and warrant further investigation.

### Limitations

Several limitations of this study should be acknowledged. First and foremost, the absence of healthy control iPSC-derived organoids precludes the ability to determine whether the observed SCFA-induced transcriptional changes are specific to ASD or represent general responses of neural tissue to SCFA exposure. Consequently, our findings should be interpreted as descriptive of SCFA effects in an ASD model system rather than as evidence of disease-specific mechanisms or therapeutic potential. Second, although brain organoids recapitulate certain aspects of early human neurodevelopment, they lack functional vasculature, immune cells (e.g., microglia), and mature synaptic circuitry, which limits their ability to model complex neuroinflammatory and gut–brain axis interactions. Third, the relatively short duration of SCFA exposure (7 days) may not capture long-term adaptive or compensatory transcriptional responses. Future studies incorporating healthy control organoids, longer exposure protocols, broader concentration ranges, and more complex model systems (e.g., assembloids or in vivo models) are necessary to validate and extend our observations.

## Conclusion

In conclusion, this transcriptome sequencing study characterized global gene expression changes in ASD-derived cerebral organoids following exposure to acetate or butyrate. Butyrate treatment was associated with enrichment of the TGF-β signaling pathway, while acetate treatment primarily affected genes related to cellular permeability and membrane functions. These findings identify candidate molecular pathways through which SCFAs may act in an ASD model system. However, due to the absence of healthy control organoids, the current data do not permit conclusions regarding ASD-specific effects or therapeutic potential. Future studies incorporating control organoids and in vivo models are necessary to validate these observations and to determine whether the identified pathways represent disease-relevant mechanisms.

## Supporting Information

S1 Fig CCK-8 viability assay for sodium acetate treatment in ASD cerebral organoids. Organoids were treated with increasing concentrations of sodium acetate (0, 10, 50, 100, 200, 500, 1000 μM) for 24 hours. Cell viability is expressed relative to the untreated control (0 μM, set to 1.0). Data are presented as mean ± SD (n = 3 biological replicates). No significant reduction in viability was observed at any concentration tested. The concentration of 100 μM (indicated by arrow) was selected for subsequent transcriptome sequencing experiments.
(TIF)

S2 Fig CCK-8 viability assay for sodium butyrate treatment in ASD cerebral organoids. Organoids were treated with increasing concentrations of sodium butyrate (0, 10, 20, 50, 100, 200, 500, 1000 μM) for 24 hours. Cell viability is expressed relative to the untreated control (0 μM, set to 1.0). Data are presented as mean ± SD (n = 3 biological replicates). Viability remained above 90% at concentrations ≤100 μM but declined to approximately 70% at 200 μM and 45% at 1000 μM. The concentration of 100 μM (indicated by arrow) was selected as the highest non-toxic dose for subsequent transcriptome sequencing experiments.
(TIF)
