# Supplementary figures and images for: Differential analysis of Short chain fatty acids incubation in autistic organoids based on transcriptome sequencing
Source: PLoS One. 2026 Jun 5;21(6):e0351146. doi: 10.1371/journal.pone.0351146 (PMC13240876; doi:10.1371/journal.pone.0351146)

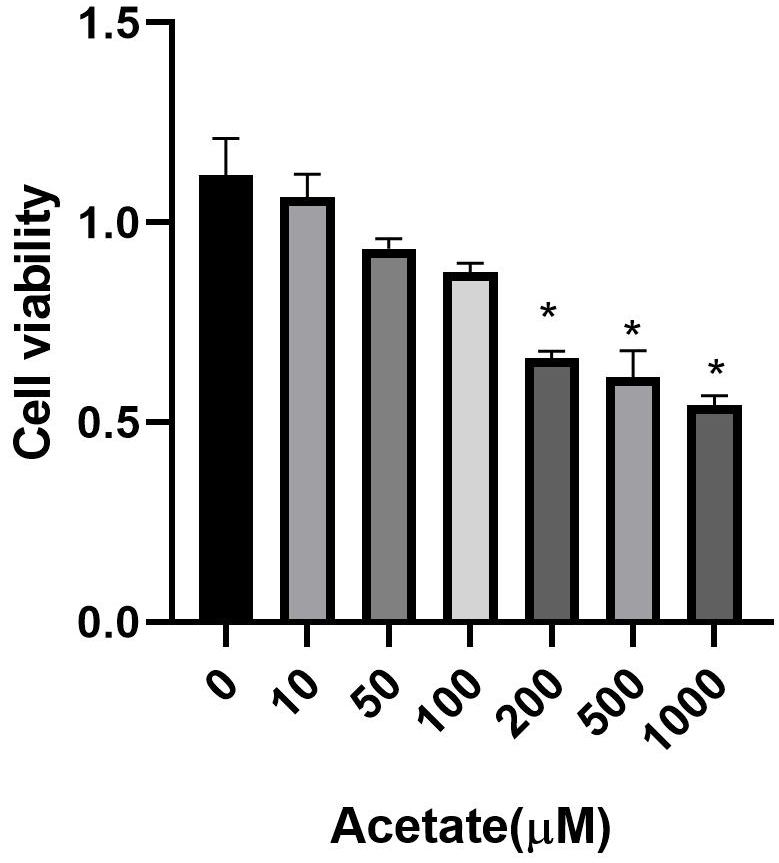

Supplement: S1 Fig — Organoids were treated with increasing concentrations of sodium acetate (0, 10, 50, 100, 200, 500, 1000 μM) for 24 hours. Cell viability is expressed relative to the untreated control (0 μM, set to 1.0). Data are presented as mean ± SD (n = 3 biological replicates). No significant reduction in viability was observed at any concentration tested. The concentration of 100 μM (indicated by arrow) was selected for subsequent transcriptome sequencing experiments. (TIF) [file pone.0351146.s001.tif]

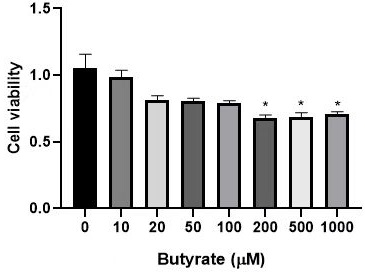

Supplement: S2 Fig — Organoids were treated with increasing concentrations of sodium butyrate (0, 10, 20, 50, 100, 200, 500, 1000 μM) for 24 hours. Cell viability is expressed relative to the untreated control (0 μM, set to 1.0). Data are presented as mean ± SD (n = 3 biological replicates). Viability remained above 90% at concentrations ≤100 μM but declined to approximately 70% at 200 μM and 45% at 1000 μM. The concentration of 100 μM (indicated by arrow) was selected as the highest non-toxic dose for subsequent transcriptome sequencing experiments. (TIF) [file pone.0351146.s002.tif]
